# COVID-19-Related Pneumonia in an Adolescent Patient with Allergic Asthma

**DOI:** 10.1155/2021/6706218

**Published:** 2021-10-07

**Authors:** Öner Özdemir, Muhammet Mesut Nezir Engin, Emine Aylin Yılmaz

**Affiliations:** ^1^Professor of Pediatrics, Division of Allergy and Immunology, Adapazarı, Sakarya, Turkey; ^2^Physician in Pediatrics, Department of Pediatrics, Research and Training Hospital of Sakarya University Medical Faculty, Adapazarı, Sakarya, Turkey

## Abstract

**Background:**

The latest coronavirus infection due to SARS-CoV-2, which started in China in December 2019, was announced as a pandemic by the World Health Organization (WHO) in March 2020. All epidemiological data so far show us that SARS-CoV-2 infection is less serious in children than in adults. Allergic asthma, the most common chronic disease in children, is usually not to be related to greater risk or severity for COVID-19 in pediatric populations. Although reports/research on asthma and COVID-19 in children have thus far been comforting, when coming across an asthma patient with any lower airway infection, attention should be given to evaluate their asthma control level and the possibility of SARS-CoV-2 infection. *Case Report*. Here, we report a rare adolescent case of COVID-19-related pneumonia development with underlying asthma. A 16-year-old male patient has been followed up by the pediatric allergy outpatient clinic with the diagnosis of asthma for the last 5 years. He was thought to have typical clinical and laboratory findings for SARS-CoV-2 infection combined with underlying pediatric (allergic) asthma. Pulmonary CT showed findings consistent with COVID-19-related pneumonia. He was discharged after 1 week when all his complaints regressed, his examination became normal, and 5-day favipiravir treatment was completed.

**Conclusion:**

When a physician comes across an asthma patient with any lower airway infection, attention should be given to evaluate their asthma control level and possibility of SARS-CoV-2 infection.

## 1. Introduction

Coronavirus pandemic, owing to severe acute respiratory syndrome coronavirus 2 (SARS-CoV-2) infection, which started in Wuhan, China, in December 2019, was later announced as a pandemic by the World Health Organization (WHO) in March 2020. SARS-CoV-2 is the third highest known pathogen coronavirus, whose mortality rates range from 6 to 10.5%, depending on the comorbidity of the infected individuals [1, 2]. At least, a quarter of coronavirus disease 2019 (COVID-19) patients are reported to have one of the chronic diseases as a comorbidity [3]. Some of the comorbidities, especially chronic respiratory tract diseases (such as asthma and COPD) with long-standing drug treatments, may influence the development, therapy, and prognosis of COVID-19 [4].

Here, a rarely seen case of a 16-year-old adolescent male patient is discussed under the light of the current literature, since he developed severe COVID-19-related pneumonia with the preexisting diagnosis of asthma.

## 2. Case Report

A 16-year-old male patient has been followed up by the pediatric allergy outpatient clinic with the diagnosis of asthma for the last 5 years. The patient was regularly using daily montelukast 10 mg tablet and budesonide + formoterol 80/4.5 mcg 2x1 puffs in the maintenance treatment of asthma. His persistent asthma has been under control, and its severity is mild. His pulmonary function test (spirometry) was found to be within normal limits (predicted FEV1: 131%, FVC: 136%, FEV1/FVC: 92, and PEF: 90%) several times for the last 3 years. He was detected to be allergic to molds and wild grasses. A skin prick test showed indurations as negative control: 0 × 0 mm, positive control: 6 × 6 mm, *Chenopodium album*: 3 × 3 mm, and *Cladosporium*: 3 × 3 mm.

He had symptoms of persisting fever up to 39°C degrees in the morning and night for 5 days and cough with green sputum and described a very severe headache for 3-4 days in the last week. He assessed the severity of headache as 10/10 with a visual analog scale. Also, the patient was experiencing orthopneic dyspnea while coughing. It was learned that he had previous contact with a SARS-CoV-2-positive individual.

**Figure 1:**
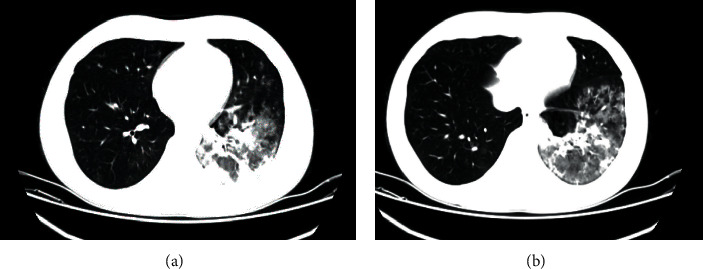
The patient's thorax CT images show radiologic findings consistent with COVID-19 pneumonia. Diffuse ground glass-like consolidated nodular lesions in the left hemithorax continue to the lower lobes.

In the initial physical examination, he seemed to have mild dyspnea and tachypnea, body temperature was 39.5°C, heart rate: 110/min, respiratory rate: 38/min, and oxygen saturation: 88% (without oxygen support). Pulmonary auscultation revealed diffuse sibilant and subcrepitant rales on the anterior and posterior parts of both the lungs and on the left basal region at the end of inspiration and beginning of expiration. Rest of the physical examination was within the normal limit. According to the laboratory results, initial leukocyte, lymphocyte, eosinophil, and platelet counts were decreased. In his routine biochemistry, AST and ALT levels were increased (Table 1). Prognostic biomarkers for COVID-19 such as CRP, LDH, ferritin, D-dimer, and fibrinogen levels were mildly elevated. PCR tests for SARS-CoV-2 were detected by nasopharyngeal swabs as positive twice. Serum immunoglobulins were within ±2 standard deviations of normal values. Pulmonary CT showed findings consistent with COVID-19-related pneumonia. Diffuse ground glass-like consolidated nodular lesions in the left hemithorax of the patient continued to the lower lobes. Ground glass-like nodular lesions were also observed in the peripheral areas in the upper lobe and in the superior segment of the lower lobe of the right lung (Figure 1(a)).

He was thought to have typical clinical and laboratory findings for SARS-CoV-2 infection combined with underlying pediatric (allergic) asthma. Final diagnosis is confirmed as COVID-19-related pneumonia with preexisting asthma. After diagnosed with COVID-19 pneumonia, he was admitted to the pediatric COVID-19 inpatient service. In addition to maintenance fluid, favipiravir (2x8 tablets on the first day and 2x3 tablets for the rest of 4 days, po) and methylprednisolone (2 mg/kg/day, iv route) were given for COVID-19. Also, for pneumonia, ceftriaxone (50 mg/kg/day, 3 gr/day, iv), clarithromycin (15–20 mg/kg/day, 1 gr/day, po), and paracetamol (3-4x15 mg/kg/dose, po) were administered. Since the patient sometimes had up to 39.5°C fever and described an increase in cough and dyspnea during the admission, inhaled corticosteroid 80 mcg 4x1 puffs was added to his treatment on the 4^th^ day of admission. Without oxygen support, oxygen saturation (SpO2) level was usually observed at >95%. He was discharged after 1 week when all his complaints regressed, his examination became normal, and 5-day favipiravir treatment was completed. He was later followed up by the pediatric allergy outpatient clinic. During follow-ups, his repeated spirometry values were within normal limits and he did not need any physical therapy (**we have obtained an informed consent form from the patient's parents for publication of this case report**).

## 3. Discussion

We are reporting a rare case of suffering from COVID-19-related pneumonia with preexisting pediatric allergic asthma. As described above in our case with asthma, SARS-CoV-2 positivity in PCR testing and findings of typical ground glass-like opacity in chest CT were consistent with COVID-19 [5–7].

Because COVID-19 primarily affects the lungs, certain diseases (such as congenital heart disease, diabetes mellitus, and some primary immune deficiency diseases) have been identified as a potential risk factor for COVID-19 [8]. However, asthma has not been generally thought as a specific risk factor for severe COVID-19 development [9–11]. While some meta-analyses reported almost no data related to asthma patients, some others reported that asthma was thought to be associated with an increased risk of COVID-19 mortality in hospitals. Where do asthmatic patients stand in this pandemic? Are asthmatic patients more inclined to be affected by SARS-CoV-2 infection? When afflicted, are asthmatic children at greater risk of developing severe COVID-19? Another issue is if the association between asthma and COVID-19 progress differs according to the age of the patient (adults vs. children) [12–14].

According to his initial laboratory results, leukocyte, lymphocyte, eosinophil, and platelet counts decreased, which were related to myelosuppression of SARS-CoV-2 infection (Table 1). According to the laboratory results, all his immunoglobulin levels were found to be normal except for total IgE. Overall, although these clinical and laboratory findings demonstrated the severity of the disease, the patient overcame the disease without need of oxygen support and intensive care unit treatment. This course was also consistent with a report of Turkish pediatric patients by Karbuz et al. [15]. Similar to our case, this report describes that pediatric asthma patients may have a symptomatic course but do not require mostly intensive care admission. In this report, cough was the key symptom in asthmatic cases (80.0%), and 87.6% of children with asthma were symptomatic (*p*: 0.016). Nevertheless, being asthmatic was not always associated with disease gravity [15]. After 5 days of favipiravir treatment, liver function test (AST and ALT) levels of our patient were found to be doubled. This may be due to the use of the hepatotoxic effect of paracetamol and/or favipiravir [16].

In many nations, such as China, Brazil, India, Mexico, Saudi Arabia, Spain, and Italy, the asthma prevalence in COVID-19 cases was lesser than that detected in the overall population, while the contradictory findings were demonstrated in the USA, Australia, UK, and Ireland [14]. In a Chinese study, only one of the 182 pediatric cases with COVID-19 was admitted in Wuhan had asthma [17]. On the other hand, in Brazil and New York, it was demonstrated that asthma was a common comorbidity, but was not linked with more severe COVID-19 leading to hospitalization or the need for intensive care therapy [6]. In an online survey of 91 asthma specialists, caring for more than 133,000 pediatric asthma cases in 5 continents, only 14% of the responders described assumed COVID-19 patients among their cases; asthmatic children had mild symptoms in 73% of them, and only 1 patient needed hospitalization [18].

Respiratory viruses are one of the recognized triggers of asthma attacks. Conventional coronaviruses are respiratory viruses and have been accused of both upper airway infections and asthma attacks upon infection. Reported entry receptors for most conventional coronaviruses do not include ACE2 (angiotensin-converting enzyme-2) [19]. Even for asthma patients, it is difficult to distinguish respiratory COVID-19 symptoms such as chest tightness, coughing, and shortness of breath from an asthma exacerbation by other triggers. Consequently, it is crucial to draw attention to the surveillance and active management of chronic preexisting diseases in COVID-19 cases. In a study, mild bronchospasm was noticed in 24% of the cases, and oral steroids were used in only 1 case [20]. In reality, COVID-19 has not been held responsible as an essential driver of viral wheezing or asthma attacks in children [21].

Favipiravir as an antiviral agent and anti-inflammatory methylprednisolone were used in his treatment. Corticosteroid therapy is the main treatment for asthma, but its use in viral pneumonia is debatable. Steroids are supposed to be able to antagonize some pathophysiological developments of acute respiratory distress syndrome, comprising extreme inflammatory response [4]. According to an assessment of the Cochrane system, glucocorticoids can lessen the fatality of cases with severe community-acquired pneumonia as well [22]. There is a complex relationship among asthma, inhaled corticosteroid use, and ACE2 expression. According to Severe Asthma Research Program-3 (SARP-3), the use of inhaled corticosteroids is linked with a decreased level of ACE2 and transmembrane protease serine 2 (TMPRSS2) gene expressions in asthmatic patients which are the key entrance receptors for SARS-CoV-2 infection [23]. Also, all guidelines and experts suggest that asthma patients continue taking their inhaled corticosteroids using a metered-dose-inhaler (MDI) during this pandemic [24, 25].

Some of the potential mechanisms suggested in the relevant literature for a milder COVID-19 course in asthmatic children are as follows. Seriousness and complications of any SARS-CoV-2 infection are linked with the production of hyperinflammation. In asthmatics, such hyperinflammation could be decreased by several factors including the hindered and ineffective antiviral reaction because of lessened IFN-*α* production by dendritic and epithelial cells. Nevertheless, the defensive role of eosinophilic inflammation, Th2 type inflammatory mediator (e.g., IL-4/-5/-13) predominance in the respiratory system, and antiviral and immunomodulatory properties of inhaled corticosteroids are also thought to play a strong role. Asthma and respiratory allergy, as a type 2 eosinophilic inflammation, and allergen exposures are connected with decreased expression of the ACE2 gene in bronchial epithelial cells. Eosinophils also might help in the reduction of viral load with subsequent attenuation of SARS-CoV-2 infection [26–28].

Due to stay-at-home orders, physical activity is restricted and the risk of contact with COVID-19-positive parents is greatly increased. The longer the stay at home, the greater the risk of further exposure to indoor allergens such as mold, rodents, cockroaches, and tobacco smoke. Indoor air quality and air conditioning will be more essential during stay-at-home times. Therefore, indoor plants where asthmatic children live are also very important as potential attack triggers.

Our strength in this report is that this is a rare allergic asthma case developing severe COVID-19-related pneumonia even under control with maintenance therapy. Our limitation is this is just one case, and it is hard to make definitive conclusion and does not reflect the whole reality on COVID-19 progress in these allergic asthma cases, especially controlled asthma cases.

## 4. Conclusions

Although reports/research on asthma and COVID-19 in children have so far been comforting [29], the European Academy of Allergy and Clinical Immunology (EAACI) stated that “reliant on good judgment, rather than growing data” pediatric patients with asthma, particularly if severe or uncontrolled ones, should be contemplated to be at higher risk of developing severe COVID-19.

A take-home message from this case is when physicians come across an asthma patient in clinic with any lower airway infection, attention should be given to evaluate their asthma control level and possibility of SARS-CoV-2 infection [30, 31]. Nevertheless, even asthma patients under control could develop SARS-CoV-2-associated pneumonia.

## Figures and Tables

**Table 1:** Laboratory results of the patient are shown.

Parameters	Day 1	Day 4	Day 7	Reference ranges
WBC (/mm^3^)	**3600**	4730	6940	4600–10200
Hemoglobin (g/dl)	16, 3	158, 8	15, 4	12, 2–18, 1
Hematocrit (%)	50, 3	49, 7	47, 7	37, 7–53, 7
Absolute neutrophil count (/mm^3^)	2260	3520	5490	2000–6900
Absolute lymphocyte count (/mm^3^)	**993**	**1060**	**925**	600–3400
Absolute eosinophil count (/mm^3^)	5	3	**1**	<700
Platelet count (/mm^3^)	**121.000**	**122.000**	213.000	142000–424000
Urea (mg/dL)	17, 35	19, 39		17–43
Creatinine (mg/dL)	0, 69	0, 94	0, 54	0.26–0.77
AST (U/L)	26	30	**82**	0–50
ALT (U/L)	29	25	**128**	0–50
C-reactive protein (mg/L)	7, 16	19, 25	6, 21	0–5 mg/dL
Ferritin (ng/ml)	68, 5	163	187, 5	4, 6–204
Fibrinogen (mg/dl)			**425**	200–400
D-dimer (*μ*g FEU/L)	391	**576**	**588**	<500
LDH (U/L)	248, 92	396, 2	**431, 84**	180–430
PT (second)			12, 6	8–13.2
aPTT (second)			24, 8	18.5–33.5
INR			1, 2	0.8–1.3
Ig G (mg/dL)		768		639–1344
Ig M (mg/dL)		74		56–352
Ig A (mg/dL)		89, 4		70–312
Anti-B titer		1/256		
SARS-CoV-2 PCR	POSITIVE	POSITIVE		
Total Ig E		**274**		0–100 IU/ml

WBC: white blood cell; Ig: immunoglobulin; PCR: polymerase chain reaction.
